# Redox process is crucial for inhibitory properties of aurintricarboxylic acid against activity of YopH: virulence factor of *Yersinia pestis*

**DOI:** 10.18632/oncotarget.4625

**Published:** 2015-07-22

**Authors:** Alicja Kuban-Jankowska, Kamlesh K Sahu, Pawel Niedzialkowski, Magdalena Gorska, Jack A Tuszynski, Tadeusz Ossowski, Michal Wozniak

**Affiliations:** ^1^ Department of Medical Chemistry, Medical University of Gdansk, Gdansk, Poland; ^2^ Department of Physics, University of Alberta, Edmonton, Canada; ^3^ Li Ka Shing Institute of Virology, University of Alberta, Edmonton, Canada; ^4^ Department of Analytical Chemistry, Faculty of Chemistry, University of Gdansk, Gdansk, Poland

**Keywords:** Pathology Section, ATA (aurintricarboxylic acid), YopH, protein tyrosine phosphatase inhibitor, virulence factor inhibition, oxygen reduction/oxidation

## Abstract

YopH is a bacterial protein tyrosine phosphatase, which is essential for the viability and pathogenic virulence of the plague-causing *Yersinia sp*. bacteria. Inactivation of YopH activity would lead to the loss of bacterial pathogenicity. We have studied the inhibitory properties of aurintricarboxylic acid (ATA) against YopH phosphatase and found that at nanomolar concentrations ATA reversibly decreases the activity of YopH. Computational docking studies indicated that in all binding poses ATA binds in the YopH active site. Molecular dynamics simulations showed that in the predicted binding pose, ATA binds to the essential Cys403 and Arg409 residues in the active site and has a stronger binding affinity than the natural substrate (pTyr). The cyclic voltammetry experiments suggest that ATA reacts remarkably strongly with molecular oxygen. Additionally, the electrochemical reduction of ATA in the presence of a negative potential from −2.0 to 2.5 V generates a current signal, which is observed for hydrogen peroxide. Here we showed that ATA indicates a unique mechanism of YopH inactivation due to a redox process. We proposed that the potent inhibitory properties of ATA are a result of its strong binding in the YopH active site and *in situ* generation of hydrogen peroxide near catalytic cysteine residue.

## INTRODUCTION

*Yersinia* genius contains three species of bacteria pathogenic to humans: plague-causing *Yersinia pestis*, septicemia-inducing *Yesinia tuberculosis* and *Yersinia enterocolitica*, which is responsible for a range of gastrointestinal disorders [1]. *Yersinia pestis* is transmitted by fleas while *Y. tuberculosis* and *Y. enterocolitica* are transmitted by the fecal oral route [2].

*Yersinia sp*. utilizes a type III secretion system for translocation of virulence effectors into the host cell [3]. All three *Yersinia* species contain a 70kb plasmid that encodes a type III complex system and effectors (Yops). During infection, *Yersinia* translocates Yops virulence effectors into a host cell leading to inhibition of the innate immune response [4].

One of *Yersinia*'s outer membrane protein effectors is a highly active YopH protein tyrosine phosphatase, which is essential for virulence since the YopH mutant plasmid is avirulent [5]. YopH is causing deregulation of cellular functions, disrupting focal complex structures and blocking phagocytosis [6]. YopH disturbs the focal adhesions by dephosphorylation of the focal adhesion kinase (FAK) and suppresses the production of reactive oxygen species by macrophages [7].

The catalytic center of the YopH contains an amino acid sequence similar to eukaryotic protein tyrosine phosphatases (PTPs). There is a cysteine residue located at the bottom of the active site, which is essential for catalysis and enzymatic activity [8]. As for the eukaryotic PTP family, the catalytic cysteine is highly vulnerable to oxidation, which results in inactivation of the enzyme and depends on the oxidation state leads to formation in the active site, respectively, of an irreversible sulfenic acid residue or irreversible sulfinic and sulfonic acid residues [9].

*Yersinia pestis* still causes several thousand human cases per year and the climate change is increasing the risk of plague outbreaks in new geographic areas as well as spreading of its natural reservoirs [10]. For historical reasons and due to the fact that *Yersinia pestis* is one of the most virulent infectious agents threatening humans, there is an ever increasing risk of the use of *Y. pestis* by unauthorized groups as a biological weapon of terror [11]. The growing resistance of humans to antibiotics is one of the reasons to search for new treatment options and the bacterial virulence factor YopH is well positioned to become a new candidate for drug discovery [12].

There are libraries of chemical compounds that have been tested as YopH inhibitors and numerous of them were reported to inhibit YopH activity, mostly in micromolar concentrations [13]. Examples of such compounds include salicylic acid derivatives [14], natural substrate (pTyr) mimetics with carboxyl groups [15] or natural compounds, such as bromotyrosine alkaloids purified from a marine sponge [16].

**Figure 1:**
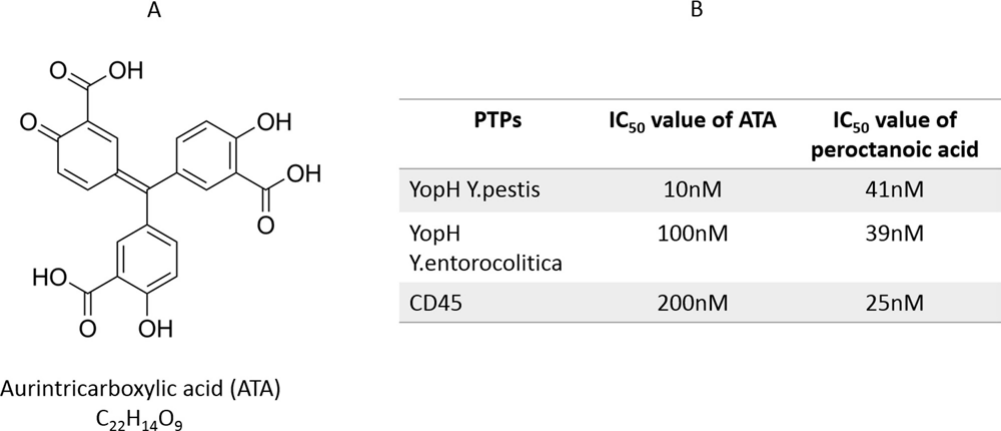
ATA as YopHs inhibitor **A.** The structure of aurintricarboxylic acid (ATA). **B.** IC_50_ values of ATA and peroctanoic acid for YopHs and CD45 inhibition. IC_50_ values were determined from a plot presenting ATA or peroctanoic acid concentration versus percentage of the enzymatic activity measured as absorbance with *p*NPP substrate of recombinant CD45, YopH after 15 minutes incubation with inhibitors.

One of the most effective YopH inhibitors is aurintricarboxylic acid (Figure 1A), with an IC_50_ value around 10 nM, which was evaluated by the Liang group [5] and confirmed by our results (Figure 1B). Aurintricarboxylic acid (ATA), a polyaromatic carboxylic acid derivative exhibiting polyanionic properties (Figure 1A), is a red dye and is not only known to inhibit protein tyrosine phosphatases but also nucleic acid binding enzymes, such as reverse transcriptase, DNA and RNA polymerase, topoisomerase and nuclease [17]. It is considered to be a potential anti-AIDS compound by preventing binding of HIV coat protein, gp120, to its CD4 receptor [17]. It has been demonstrated that ATA can inhibit the replication of viruses from several different families, such as the human immunodeficiency virus, also coronavirus, vesicular stomatitis virus and vaccinia virus. It does so by the inhibition of phosphatase activity of viral enzyme [18]. Because of its binding with cellular endonucleases, topoisomerases and various important signaling pathways, ATA has also been found to prevent apoptosis in a variety of cell models [19].

Taking the above studies into consideration, it is readily concluded that ATA is a remarkable compound, which should be seriously considered as a drug for infectious diseases, including epidemics caused by *Yersinia sp*.

We decided to evaluate the effectiveness of ATA as YopH inhibitor and to study possible mechanisms of ATA induced inactivation, as well as electrochemical properties of ATA. We performed computational docking and molecular dynamic studies to gain a molecular-level insight into the binding affinities and conformations of ATA in the YopH active site.

## RESULTS

### Inhibitory effects of ATA on YopH and CD45 enzymatic activity

YopH recombinant phosphatase from *Yersinia pestis* and *Yersinia eneterocolitica* was treated with ATA and IC_50_ values were calculated. We also treated YopHs with peroctanoic acid, containing a peroxycarboxyl group with a higher oxidizing potency than that of a carboxyl group, studied by our group as a strong PTP inhibitor [20], in order to compare the inhibitory effects. In addition, the human CD45 recombinant protein tyrosine phosphatase was utilized to study the inhibitory properties of ATA on human PTP.

We found that ATA inactivates YopH and CD45 phosphatases at nanomolar concentrations. Interestingly, considering IC_50_ values, ATA was more effective against YopH from *Y. pestis* and YopH from *Y. enterocolitica* than against CD45 phosphatase (Figure 1B). Comparing the inhibitory effect of ATA with peroctanoic acid, ATA inactivated *Y. pestis* YopH with greater potency than peroctanoic acid, but peroctanoic acid was found to be a stronger inhibitor of CD45 (Figure 1B).

### The mechanism of ATA induced inactivation of YopH

**Figure 2:**
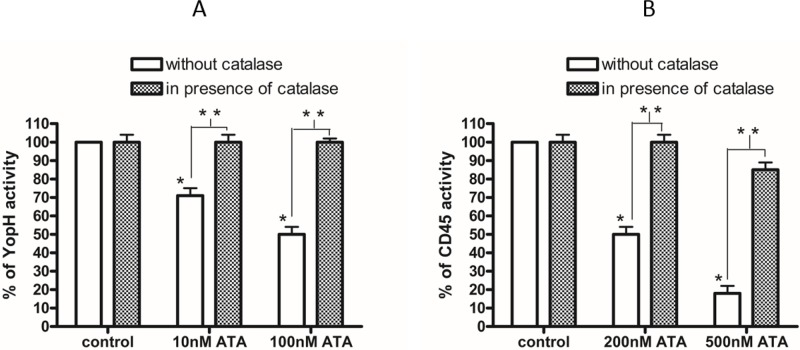
The reversibility and mechanism of ATA inhibition **A.** Reduction assay of YopH *Y. enterocolitica* activity with DTT. Recombinant YopH was pretreated for 15 minutes with 100 nM ATA and subsequently incubated with 10 mM DTT to reverse the ATA inhibition. The increase of activity of YopH was measured every minute on microplate reader as absorbance at 405 nm using *p*NPP substrate. Data presented as percent of control. **B.** The amount of modified YopH thiol adducts with NBD (Cys-S-NBD adducts) after 15 minutes of treatment with 100 nM ATA with/without catalase (500U/ml). Data presented as absorbance (420 nm), means ± SD (*n* = 3). One-way Anova test. * significantly different (*P* < 0.001).

We prepared a reduction assay with dithiothreitol (DTT) to investigate the reversibility of ATA induced inhibition. We observed that ATA inactivates YopH reversibly and the ATA induced inhibition of YopH was completely reversed after a 20 minutes incubation process with DTT (Figure 2A).

Due to the fact that YopH, as other PTPs, contains an oxidation-sensitive cysteine located in the active site, we decided to prepare an NBD-Cl assay to calculate the amount of thiol groups after treatment with ATA. We tested the amount of thiol groups modified by NBD-Cl forming Cys-S-NBD adducts. After treatment of recombinant YopH with 100 nM ATA, the quantity of Cys-S-NBD adducts was significantly decreased in comparison with control (Figure 2B). Application of ATA has resulted in an over 50% reduction of thiol-NBD adducts compared to control, suggesting that thiol groups in YopH after treatment with ATA are likely to be in oxidized form. Interestingly, the addition of catalase completely prevented ATA induced decrease of thiol adducts (Figure 2B).

**Figure 3:**
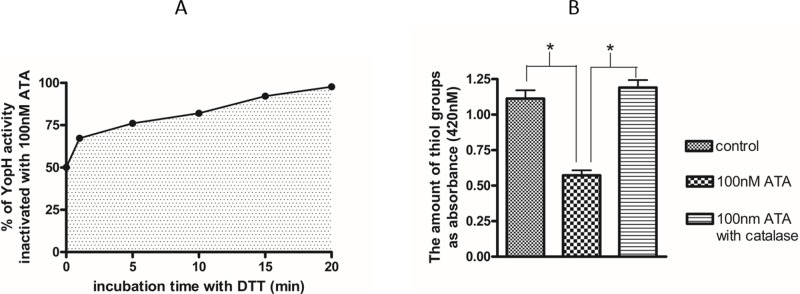
The elimination of ATA inhibitory properties by catalase **A.** Activity of YopH after treatment with 10 nM and 100 nM ATA in presence and absence of catalase (500U/ml). The enzymatic activity of YopH was measured on microplate reader as absorbance at 405 nm using *p*NPP substrate. Data presented as percent of control, means ± SD (*n* = 10). *T*-test analysis of variance. *significantly different (*P* < 0.001) from control, **significantly different (*P* < 0.001) in pairs. **B.** Activity of CD45 after treatment with 200 nM and 500 nM ATA in presence and absence of catalase. The enzymatic activity of CD45 was measured on microplate reader as absorbance at 405 nm using *p*NPP substrate. Data presented as percent of control, means ± SD (*n* = 10). *T*-test analysis of variance. * significantly different (*P* < 0.001) from control, **significantly different (*P* < 0.001) in pairs.

We also observed that catalase not only prevents a decrease of thiol adducts but also prevents an inhibition of enzyme activity. The catalase pretreatment almost completely protected from ATA induced YopH inactivation (Figure 3A), as well as CD45 inactivation (Figure 3B). The elimination of inhibitory effects of ATA in the presence of catalase and a loss of thiol adducts after ATA treatment may lead to the conclusion that ATA is probably inactivating PTPs due to an oxidative mechanism.

### Electrochemical experiments

**Figure 4:**
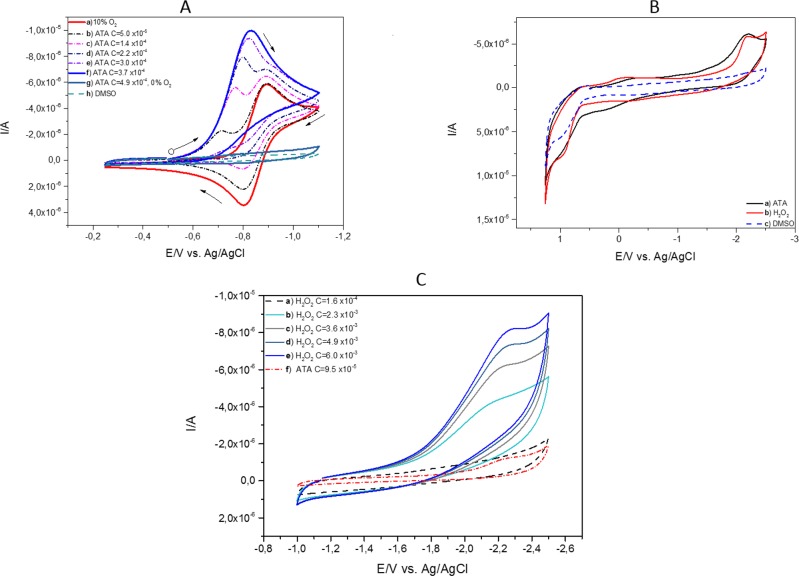
Cyclic voltammetry of ATA **A.** A series of cyclic voltammograms (CVs): a) of 2.2 · 10^−4^ M of oxygen in DMSO solutions (red line) at gradually increasing concentration of ATA: b) 5.0 · 10−^5^ M, c) 1.4 · 10^−4^ M, d) 2.2 · 10^−4^ M, e) 3.0 · 10^−4^ M, (dotted lines), f) 3.7 · 10−^4^ M (blue line), g) 4.9 · 10−^4^ M of ATA without oxygen (grey line) and h) DMSO in 0.1 M TBAP, conditions: scan rate 100 mV/s. **B.** Cyclic voltammograms (CVs) of a) ATA - concentration 4.9 · 10^−4^ M in DMSO solution (black line) b) hydrogen peroxide - concentration 1, 5 · 10^−3^ M (red line) c) potential window obtained in DMSO in 0.1 M TBAP solution (blue dotted line), conditions: scan rate 100 mV/s. **C.** Comparison of cyclic voltammograms (CVs) of a - e) hydrogen peroxide at different concentrations from 1.6 · 10^−4^ M (black dotted line) to 6.0 · 10^−3^ M (blue line) and ATA at concentration of 9.5 · 10^−5^ M, (red dotted line), conditions: scan rate 100 mV/s.

We performed electrochemical measurement in order to investigate the oxidizing potency of ATA. In this study the signals from the redox process of ATA preformed in the DMSO solution were not observed (Figure 4A, grey line) in the region of oxidation-reduction potentials of oxygen (red line). In the solution of oxygen (2.2 · 10^−4^ M) the presence of ATA causes significant changes in the reduction and oxidation process. When the concentration of ATA increased from 5.0 · 10^−5^ to 3.7 · 10^−4^ M (dotted lines) the new reduction peak has appeared and the reoxidation process for oxygen disappeared.

A small addition of ATA caused significant changes in the cyclic voltammetric curve resulting in the appearance of a new cathodic peak at potential values form 0.74 V to 0.82 V (Figure 4A) (dotted lines). The disappearance of the anodic peak indicates that oxygen radicals react with the molecule of ATA. At the same time the appearance of a new peak is observed (blue line). This peak reached a constant value at 3.7 · 10^−4^ M of ATA concentration and is higher than the current peak for oxygen reduction (red line).

The analysis of cyclic voltammograms (CVs) in a wide potential range (Figure 4B) indicates electrochemical reduction of ATA in the presence of negative potentials ranging from −2.0 V to −2.5 V. The current signal obtained for ATA is very similar to the cyclic voltammograms observed for hydrogen peroxide. The value of the current obtained for ATA at a concentration of 4.9 · 10^−4^ M measured in DMSO solution is comparable to the hydrogen peroxide at a concentration of 1.5 · 10^−3^ M performed for potentials from 1.25 V to 2.5 V. This may indicate that the hydrogen peroxide could be present during the redox process of ATA.

Comparison of reduction processes of hydrogen peroxide in an increasing range of concentrations from 1.6 · 10^−4^ M (black dotted line) to 6.0 · 10^−3^ M (blue line) (Figure 4C) measured in the ranges from −1.0 V to −2.5 V suggests that during the redox process of ATA the hydrogen peroxide could appear at very low concentrations.

### Docking studies

**Figure 5:**
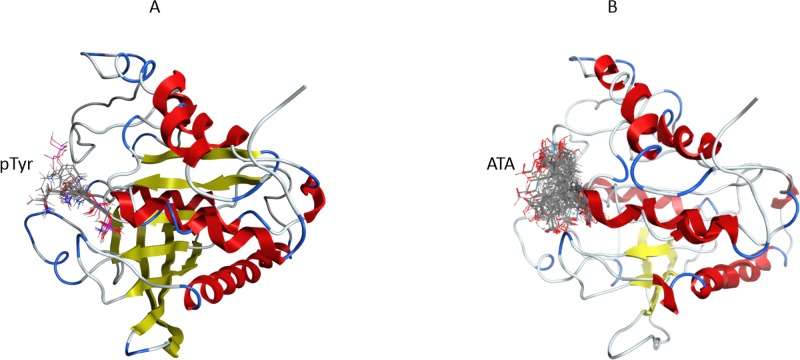
The blind flexible docking of pTyr and ATA molecules into the YopH 3D structure **A.** The site of binding for top 30 conformations of pTyr obtained from docking of pTyr into the YopH structure. In each conformation pTyr binds in the YopH active site. **B.** The site of binding for top 30 conformations of ATA obtained from docking of ATA into the YopH structure. In each conformation ATA binds in the YopH active site.

ATA and natural substrate phosphotyrosine (pTyr) molecules were docked into the 3D structure of YopH in order to investigate the possible binding conformation and affinity. We performed blind flexible docking and retained top 30 conformations from docking runs. In all 30 conformations pTyr and ATA are bound to the active site of YopH, as shown in Figure 5A for phosphotyrosine and Figure 5B for ATA. The docking studies showed that ATA can be easily accommodated inside the binding site and binds specifically in a catalytic center of YopH, in a similar manner to that of the natural substrate, phosphotyrosine (Figure 5A, 5B).

### The molecular dynamics simulations of YopH with ATA

**Figure 6:**
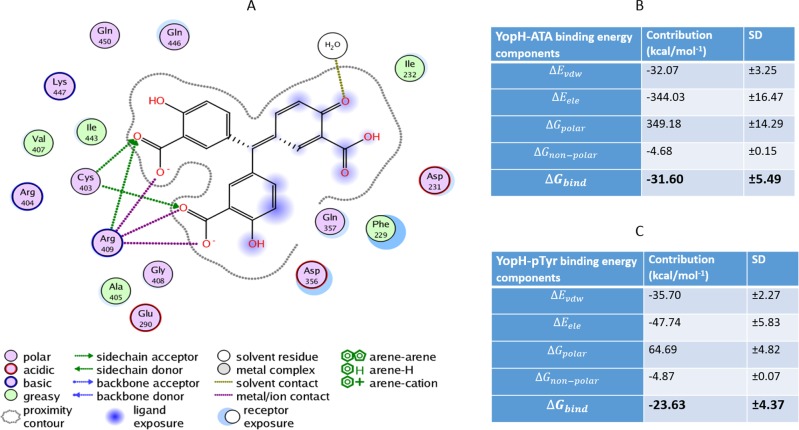
Molecular dynamic simulation of ATA in the YopH active site and binding energies for ATA and pTyr to YopH **A.** The PLIF diagram for the best binding pose of ATA in the YopH binding site. In predicted binding pose, two carboxyl groups of ATA are directed toward essential Cys403 and Arg409 residues in the active site. There are electrostatic interactions between polar groups of ATA with Cys403, Arg409 and water. **B.** Binding free energy and its components for the YopH-ATA complex by MM/GBSA methods (Kcal mol^−1^). **C.** Binding free energy and its components for the YopH-pTyr complex by MM/GBSA methods (kcal mol^−1^).

To study the binding conformation of ATA in the YopH active site we performed molecular dynamics simulations using Amber12 and identified top scoring poses from docking studies. The interactions of ATA in the YopH binding site are presented as a PLIF diagram (protein ligand interaction fingerprints) in Figure 6A. The dotted line around the molecule shows solvent contact and dotted arrows represent hydrogen bonds between amino acid residues from YopH and ATA. In the predicted binding pose two carboxyl groups of ATA are directed toward essential Cys403 and Arg409 residues in the YopH active site. Under such steric conditions there is likelihood of hydrogen bond formation between the arginine residue and carboxyl groups of ATA. As shown in the PLIF diagram, ATA has been able to utilize its polar groups to interact electrostatically with Cys403, Arg409 and water (Figure 6A). The positively charged arginine residue of the YopH active site is likely to attract the negatively charged carboxyl groups from ATA.

### The binding energies of ATA and pTyr to YopH

To indicate the binding affinities of ATA and pTyr to YopH we calculated the binding free energy for the complex. Figures 6B and 6C show the binding energies to YopH and their components for ATA and pTyr respectively. The components are described below:

Δ*E_vdw_* = the Van der Waals contribution from MM.
Δ*E_ele_* = Electrostatic energy as calculated by the MM force field
Δ*G_polar_* = The electrostatic contribution to the solvation free energy calculated by PB or GB respectively
Δ*G_non-polar_* = Nonpolar contribution to the solvation free energy calculated by an empirical model
Δ*G_bind_* = final estimated binding free energy calculated from the terms above (kcal/mol^−1^

The final estimated binding energy was calculated by the following equations:

$$\Delta G_{\mathrm{bind}}^{}=G_{\mathrm{Complex}}-G_{\mathrm{Receptor}}-G_{\mathrm{Ligand}}$$

Where G stands for free energy.

$$\Delta G_{\mathrm{bind}}^{}=\Delta E_{\mathrm{MM}}+\Delta G_{\mathrm{Polar}}+\Delta G_{\mathrm{non-Polar}}$$

Δ*G_Polar_* is the polar contribution to the solvation free energy and Δ*G_non-Polar_* is the non-polar contribution to the solvation free energy and is defined as Δ*G_non-Polar_* = *γSASA* + *b*, where *γ* = 0.0072 Kcal mol^−1^ Å^−1^ and *b* = 0 for AMBER-based GBSA calculations. SASA stands for the solvent accessible surface area. *TΔS* represents the entropic term and needs to be calculated by normal mode analysis, which is computationally expensive and since other ligands will bind to the same protein in the same binding site, we neglected the entropic contribution to the binding free energy in our calculations.

$$E_{\mathrm{MM}}=E_{\mathrm{int}}+E_{\mathrm{ele}}+E_{\mathrm{vdw}}$$

*E_MM_* is the molecular mechanics contribution to binding in vacuo expressed as a sum of the internal, electrostatic and van der Waals’ contributions. Since we use a single trajectory approach, internal energy *E_int_* will be approximately cancelled, thereby:

$$E_{\mathrm{MM}}=E_{\mathrm{ele}}+E_{\mathrm{vdw}}$$

The binding affinity indicates that ATA exhibits stronger binding to YopH than its natural substrate phosphotyrosine (Figure 6B, 6C). The comparison between binding energies of ATA and pTyr into YopH shows a much stronger van der Waals interaction for the YopH-ATA as compared to the YopH-pTyr complex.

## DISCUSSION

Earlier studies of YopH inhibitors revealed aurintricarboxylic acid as the strongest inhibitor of Yersinia YopH virulence effector [5]. In this study our objective was to evaluate the effectiveness and possible mechanisms of ATA induced inactivation of YopH. Our experiments with recombinant YopH phosphatase confirmed that ATA can effectively inactivate YopH phosphatases at nanomolar concentration.

Taking into consideration the fact that YopH has a similar amino-acid sequence in the active site as those for other PTPs [8], including human ones, we compared the effect of ATA on human CD45 phosphatase. Protein tyrosine phosphatase CD45 is abundantly expressed in all non-nucleated haematopoietic cells and is critical for T-cell and B-cell functions. It dephosphorylates the Src kinases, therefore is important to immune-receptor modulated signaling. The inactivation of CD45 would lead to serious complications for the host immunity [21]. We found that ATA is more effective against YopH phosphatase than CD45.

The reduction assay indicated that ATA is a reversible inhibitor of YopH and that the presence of catalase abolished the effect of ATA on YopH. The inactivation of the inhibitory activity of the compound in the presence of catalase is likely through an oxidative mechanism, due to the fact that catalase is an enzyme protecting cells from reactive oxygen species by decomposition of hydrogen peroxide [22]. Some of the PTP inhibitors are acting by production of hydrogen peroxide, which is suggested as a key regulator of PTP activity in cells [23]. ATA loses the inhibitory properties against PTPs in the presence of catalase, which shows that the mechanism of ATA induced inactivation of YopH is probably involved with the oxidation of catalytic cysteine in an active site. It is confirmed also by the loss of unoxidized thiol groups in YopH after treatment with ATA.

In aprotic solvents, such as dimethyl sulfoxide (DMSO) electrochemically generated superoxide anions (O_2_^−^) are solvated far less efficiently than in water [24]. This provides an opportunity to study the interactions between the reactive oxygen species and organic compounds and also to examine if organic compounds react either with an inert form of oxygen or its reduced forms. The cyclic voltammetry for generation and detection of superoxide anions by the electrochemical reduction of molecular oxygen is very useful and easy to follow the reaction of superoxide anions with bioactive compounds at the electrode surface [25]. The obtained results indicate that ATA directly reacts with oxygen and reactive oxygen forms in DMSO solution. Additionally, the electrochemical reduction of ATA negative reduction potential (−2.0 to 2.5 V) generates a current signal, which has been observed for hydrogen peroxide.

The docking studies showed that ATA bonds specifically in the active site of YopH. ATA can accommodate itself well inside the binding site as compared to pTyr and that may be the reason for a higher binding affinity of ATA to YopH and ATA as compared to pTyr. By performing molecular dynamics simulations of the complex we found that two carboxyl groups of ATA bind directly toward the essential amino-acid residues for enzyme catalysis in the active site. Cys403 and Arg409 are involved in the reaction of dephosphorylation of substrate on tyrosine residues. The binding conformation of ATA in the YopH active site contributed to the relatively strong calculated binding affinity of ATA in comparison to natural substrate.

In conclusion, we demonstrated that ATA effectively decreases the *Yersinia* YopH activity and has stronger binding to YopH active site than natural substrate. Here we proposed a unique mechanism of ATA caused inactivation of YopH involved with its redox process leading to generation of hydrogen peroxide. We suggest that the potent inhibitory properties of ATA are due to strong binding in the YopH active site and generation of hydrogen peroxide directly inside the active site. The studies on inhibitory properties of ATA against YopH are a good starting point for the synthesis of analogous inhibitors and potentially lead to clinical applications. Further studies on ATA analogs and cellular aspects of ATA are underway.

## MATERIALS AND METHODS

### Recombinant PTP YopH and CD45 activity assay

Bacterial recombinant YopH protein tyrosine phosphatase from *Yersinia pestis* was obtained from Millipore and YopH from *Yersinia enterocolitica* was obtained from Calbiochem. Human recombinant CD45 was obtained from Sigma-Aldrich. The solutions of the recombinant PTPs were prepared in 10 mM HEPES buffer pH 7.4. The final concentration of phosphatase in reaction samples was 0.8 μg/mL (10 nM). The YopHs and CD45 enzymes were untreated (control) or treated with solution of ATA and peroctanoic acid. The assay was performed in 96-well microplates, and the final volume of each sample was 200 μL. The enzymatic activities of YopHs and CD45 were measured using 1 mM chromogenic substrate *para*-nitrophenyl phosphate (*p*NPP) in 10 mM HEPES buffer pH 7.4, at 37°C. Phosphatase hydrolyzed *p*NPP to *para*-nitrophenol and inorganic phosphate. *Para*-nitrophenol is an intensely yellow colored soluble product under alkaline conditions. The increase in absorbance (due to *para*-nitrophenol formation) is linearly proportional to enzymatic activity concentration (with excessive substrate, i.e. zero-order kinetics) and was assessed at 405 nm on a microplate reader Jupiter (Biogenet) using DigiRead Communication Software (Asys Hitech GmbH).

### Reduction assay with DTT

Subsequently, recombinant phosphatase YopH that had been previously inactivated by ATA, was then treated with 10 mM dithiothreitol (DTT), and the samples were incubated at 37°C to reverse the inactivation, if possible. Restoration of YopH enzymatic activity was measured every minute as an increase of absorbance taken at 405 nm as previously described.

### YopH thiol adduct assay

The recombinant phosphatase YopH was inactivated by ATA and the amount of modified YopH thiol adduct with NBD (Cys-S-NBD adduct) was measured after 30 minutes incubation with NDB-Cl (0.6 mM in a 0.5 mL sample) as absorbance at 420 nm with a spectrophotometer.

### Electrochemical analysis

Cyclic voltammetry (CV) experiments were performed using an Autolab potentiostat/galvanostat (model PGSTAT30) supported by the GPES software. The experiments were performed at room temperature in DMSO solution with 0.1 M tetrabutylammonium tetrafluoroborate (TBAP) as the supporting electrolyte, at a scan rate of 100 mV/s under argon atmosphere or in the oxygen-saturated DMSO solution. A conventional three-electrode configuration consisting of a glassy carbon electrode (GC), a working electrode with a 3-mm diameter. The platinum wire used as the counter electrode and an Ag/AgCl in 3M KCl reference electrode were used, respectively. Each time before use, the working GC was polished with Al_2_O_3_ powder on a wet pad, then the electrode was washed with nanopure water, and dried under a stream of nitrogen. The oxygen-saturated DMSO solution used to perform measurement was obtained by the procedure described in the literature [26]. The concentration of oxygen in oxygen-saturated DMSO at 298 K was 2.2 · 10^−3^ M [27]. During all the experiments with oxygen the air was carefully excluded from the measurement cell and the oxygen was bubbled directly into the cell in order to obtain desirable concentration. The error in oxygen concentration of all measurement is estimated to be approximately 1%. The oxygen flow was adjusted by a mass flow controller (HiTec, Bronkhorst).

### Docking studies

The initial structure of YopH was imported from the RCSB protein data bank (http://www.pdb.org) with code 2YDU.pdb [28]. The structure was minimized using taff.ff forcefield of the Molecular Operating Environment software (MOE, chemical computing group). Chain A of this pdb file contains 306 residues. The ligand was removed from this pdb file and aurintricarboxylic acid (ATA) and phosphotyrosine (pTyr) were docked into the structure of YopH. A blind flexible docking simulation was performed, where the binding site was assumed to be the entire protein. The side chains were kept free to move during forcefield refinement. Alpha PMI is the placement method used with default settings (sample per conformation = 10, maximum poses = 250). London dG rescoring was used with Alpha PMI placement. Termination criteria for forcefield refinement were set as gradient = 0.001 and interactions = 500.

### Molecular dynamics simulations

Top scoring poses from docking that interacted with Cys403 were retained for molecular dynamics simulations using amber12. We allowed Leap module of Amber [29] to add missing hydrogen atoms and heavy atoms using the Amber force field (ff10) parameters [30]. To neutralize the charge of the system, we added sodium/chloride ions. The model was immersed in a truncated cubical shell of TIP3P water [31]. A time step of 2 fs and a direct-space non-bonded cutoff of 10 Å were used. After the protein preparation, all systems were minimized to remove the steric clashes that occurred. The systems were then gradually heated from 10 to 300 K over a period of 50 ps and then maintained in the isothermal–isobaric ensemble (NPT) at a target temperature of 300 K and a target pressure of 1 bar using a Langevin thermostat [32, 33] and a Berendsen barostat with a collision frequency of 2 ps and a pressure relaxation time of 1 ps, respectively. We constrained hydrogen bonds using the SHAKE algorithm [34]. We have used the velocity-Verlet algorithm (default algorithm for the Amber MD package) for MD simulations. Particle mesh Ewald (PME) procedure was used to treat long-range electrostatic interactions using default parameters [35]. After bringing the systems at our suitable temperature and pressure of 300 K and 1 bar, respectively and equilibrating the system for 500 ps, the production run was continued for 20 ns in the isothermal–isobaric ensemble at the target temperature of 300 K and target pressure of 1 bar using the same Langevin thermostat and Berendsen barostat. The structures in the trajectories were collected at 10 ps intervals. The analysis of trajectories was performed with the Ptraj module of Amber.

### Binding affinity calculations

For the binding free energy calculations, we used the standard MM/GBSA method [36]. MMPBSA.py python script was used for MM/GBSA calculations [37]. Before the MM/GBSA analysis, all water molecules and the sodium ions were excluded from the trajectory. The dielectric constant used for the solute and surrounding solvent was 1 and 80, respectively. During the analysis of the MM/GBSA trajectory, snapshots were gathered at 10 ps intervals from the last 500 ps of the 20 ns trajectory.

### Statistical analysis

The experiments were performed at least three times. The data were applied and analyzed with GraphPad Prism (GraphPad Software v.4). Statistical analyses were performed using ANOVA combined with Tukey's test or T test combined with Wilcoxon test. The data were expressed as means ± SD. Differences between means were considered significant for *P* < 0.05.
